# Supplementary material for: Non-apoptotic enteroblast-specific role of the initiator caspase Dronc for development and homeostasis of the Drosophila intestine
Source: Sci Rep. 2021 Jan 29;11:2645. doi: 10.1038/s41598-021-81261-0 (PMC7846589; doi:10.1038/s41598-021-81261-0)
Supplement: Supplementary file 1 — Supplementary Information. [file 41598_2021_81261_MOESM1_ESM.pdf]

Supplementary Information

Non-apoptotic enteroblast-specific role of the initiator caspase Dronc for development and homeostasis of the *Drosophila* intestine

Jillian L. Lindblad, Meghana Tare, Alla Amcheslavsky, Alicia Shields and Andreas Bergmann

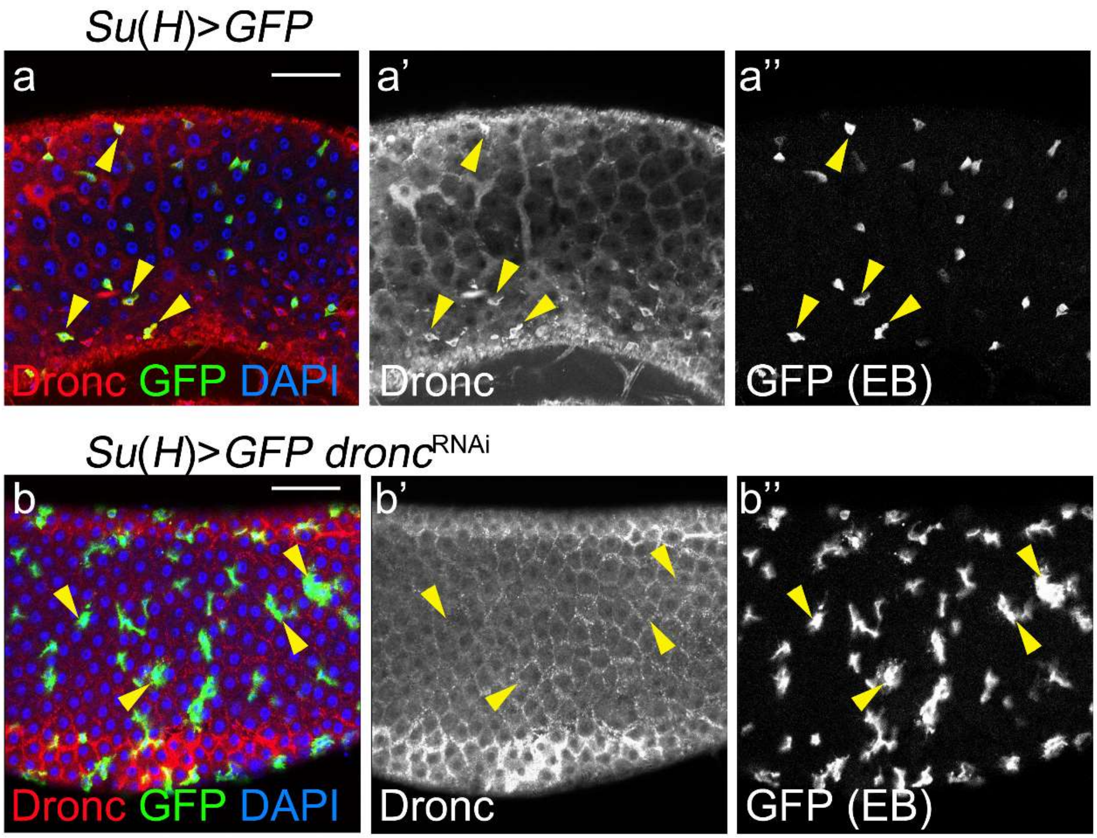

Supplementary Figure S1. Specificity control of anti-Dronc antibody. (related to Figure 2)

Anti-Dronc labeling of control and *Su*(*H*)>*dronc* RNAi intestines. Anti-Dronc labeling is red in (a,b) and grey in (a',b'). Enteroblasts (EBs) are marked by GFP (green in a,b; grey in a'',b''). DAPI (blue) marks nuclei. Yellow arrowheads in (a) highlight a few example EBs which contain Dronc protein. Yellow arrowheads in (b) highlight a few EBs which lack Dronc protein due to *dronc* RNAi. Dronc is expressed in other cells which do not express *dronc* RNAi. Scale bar: 50 µm. *dronc* RNAi line 1 = P{GD12376}v23033 was used.

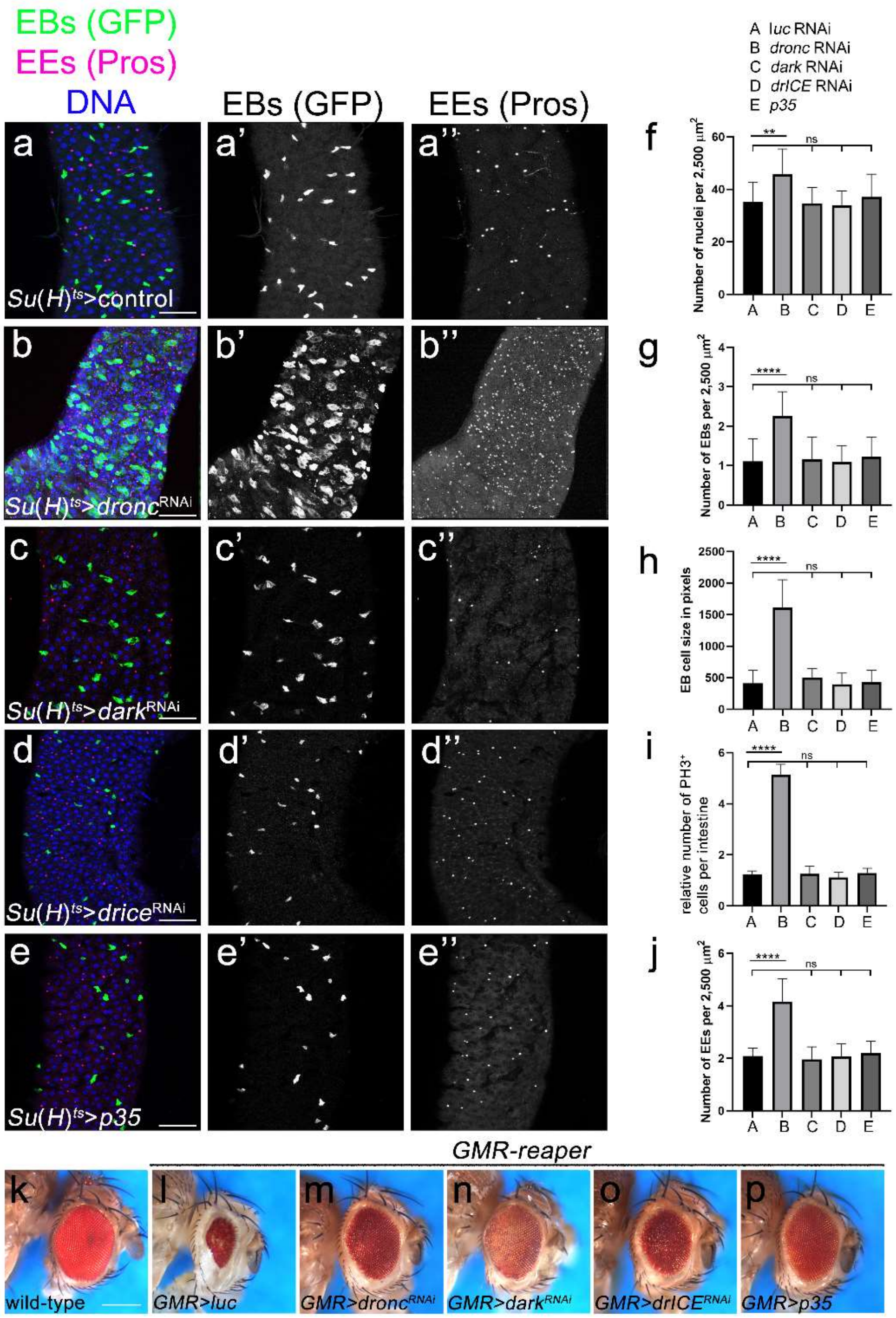

Supplementary Figure S2. *dark*^{RNAi}, *drICE*^{RNAi} and *p35* expression in EBs do not phenocopy the *dronc*^{RNAi} phenotypes in the adult midgut. (related to Figure 3)

Crosses were incubated at 18°C until adults eclosed. 5 days old females of the indicated genotype were shifted to 29°C and incubated for 5-6 days until dissection and labelling of the midguts. For comparison, the results of *dronc* RNAi are shown in (b) and quantified in (f-j). The *dronc* RNAi transgene used in (b) was P{GD12376}v23033. *Luciferase* RNAi was used as a control in (a).

(**a-e**) Shown are R4ab regions of midguts of the indicated genotypes labelled for enteroblasts (EBs) (GFP; green in a-e, grey in a'-e')), enteroendocrine (EEs) cells (Pros; red in a-e, grey in a''-e'') and nuclei (DAPI; blue in a-e). Scale bar: 50 µm. Complete genotypes: *Su(H)-Gal4 UAS-GFP tub-Gal80*^{ts}; *UAS*-X (X = *UAS-luciferase* RNAi (a), *UAS-dronc* RNAi (b), *UAS-dark* RNAi (c), *UAS-drICE* RNAi (d), *UAS-p35* (e).

(**f-i**) Quantification of the numbers of nuclei (f), EBs (g), EB cell size (h), PH3 counts (i) and number of EEs (j) of the midguts in (a-e). To obtain nuclei counts, at least five random, but representative fields of 2,500 μm^2 each per posterior midgut (region R4ab) were counted. In the case of EBs and EEs, all GFP-positive cells (corresponding to EBs) and Pros-positive cells (EEs) were counted in region R4ab and normalized to 2,500 μm^2 fields. For EB cell size calculation, the shape of EBs was outlined using the quick selection tool in Photoshop and the number of pixels in the selected area was recorded. PH3 counts were performed across the entire intestine. Data were analysed by one-way ANOVA with Holm-Sidak test for multiple comparisons. Error bars are SEM. ****$p < 0.0001$; **$p < 0.01$. ns – not significant. Number of midguts in (f,g,j) analysed: n=12 (control), 9 (*dronc* RNAi), 10 (*dark* RNAi), 11 (*drICE* RNAi), 10 (*p35*). For EB cell size calculation (h): n=19 (control), 13 (*dronc* RNAi), 34 (*dark* RNAi), 25 (*drICE* RNAi), 18 (*p35*). Number of intestines for PH3 counts (i): n=9 (control), 7 (*dronc* RNAi), 8 (*dark* RNAi), 9 (*drICE* RNAi), 7 (*p35*). Exact genotypes as in (a-e).

(**k-p**) Validation of the RNAi and *p35* transgenic lines used in (a-e). The RNAi and *p35* lines used in (a-e) were tested in the apoptosis model *GMR-reaper* which causes an eye ablation phenotype (l) compared to wild-type (Canton S) (k). The *dronc*, *dark* and *drICE* RNAi lines as well as the *p35* line driven by *GMR-Gal4* suppressed the small eye phenotype of *GMR-reaper* (m-p) suggesting that these transgenes are functional. Exact genotypes: (k) wild-type (Canton S); (l-p) *GMR-reaper GMR-Gal4*/*UAS*-X (X = *UAS-luciferase* RNAi (l), *UAS-dronc* RNAi (m), *UAS-dark* RNAi (n), *UAS-drICE* RNAi (o), *UAS-p35* (p).

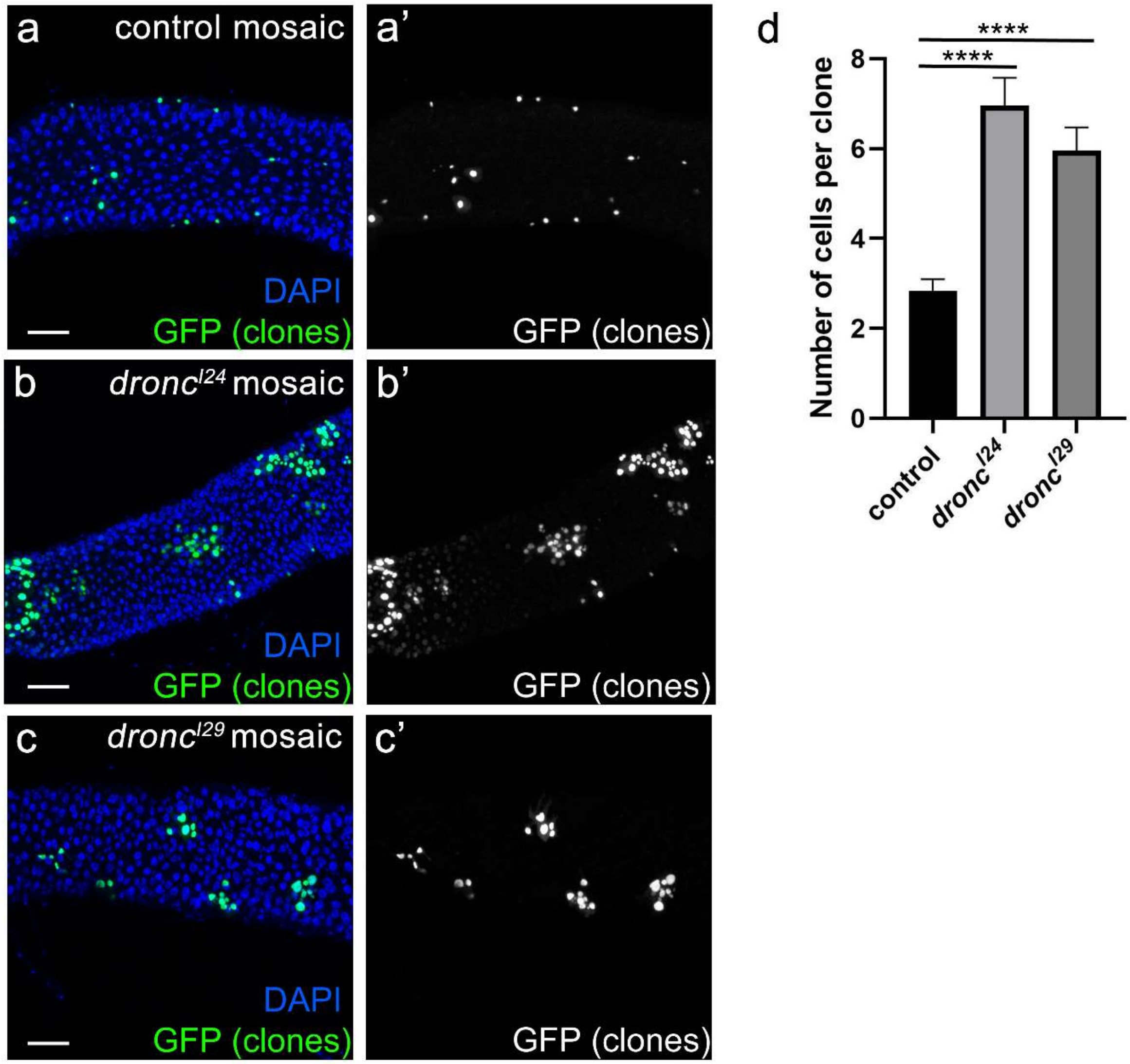

Supplementary Figure S3. Mosaic analysis of *dronc* mutants in the intestine. (related to Figure 3)

(**a-c**) Control (a) and *dronc* mutant clones using the alleles *dronc*^{I24} and *dronc*^{I29} (b,c) were induced using the MARCM technique (see Methods) and are positively labeled by GFP (green in a-c, grey in a'-c'). DAPI labeling (blue) outlines the shape of the intestine. Scale bars: 50 µm.

(**d**) Quantification of the clone sizes in (a-c). Number of cells per clone were manually counted and analyzed by one-way ANOVA with Holm-Sidak test for multiple comparisons. Error bars are SEM. ****$p < 0.0001$. n=29 (control), 23 (*dronc*^{I24}), 22 (*dronc*^{I29}).

Genotypes: (a) *yw hs-FLP/w*; *tub-Gal4 UAS-GFP*^{S65T}/+; *w*$^{+}$ *FRT80B*/*tub-Gal80 FRT80B*.
(b) *yw hs-FLP*/w; *tub-Gal4 UAS-GFP*^{S65T}/+; *dronc*^{I24} *FRT80B*/*tub-Gal80 FRT80B*.
(c) *yw hs-FLP*/w; *tub-Gal4 UAS-GFP*^{S65T}/+; *dronc*^{I29} *FRT80B*/*tub-Gal80 FRT80B*.
